# Supplementary material for: Flexible learning of quantum states with generative query neural networks
Source: Nat Commun. 2022 Oct 20;13:6222. doi: 10.1038/s41467-022-33928-z (PMC9584912; doi:10.1038/s41467-022-33928-z)
Supplement: Supplementary file 1 — Supplementary Information [file 41467_2022_33928_MOESM1_ESM.pdf]

SUPPLEMENTARY INFORMATION: FLEXIBLE LEARNING OF QUANTUM STATES WITH GENERATIVE QUERY NEURAL NETWORKS

Supplementary Note 1 Implementation details of GQNQ

A. Structure of GQNQ

As shown in Supplementary Fig. 1, our proposed Generative Query Network for quantum state learning (GQNQ) is mainly composed of a representation network $f_{\boldsymbol{\xi}}$, an aggregate function $\mathcal{A}$ and a generation network $g_{\boldsymbol{\eta}}$.

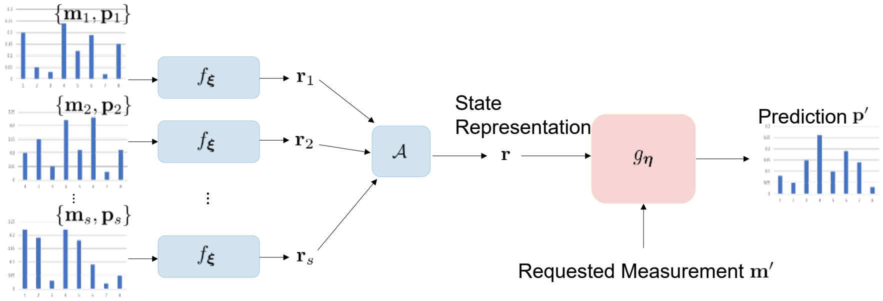

Supplementary Figure 1: Structure of GQNQ.

The representation network $f_{\boldsymbol{\xi}}$ consists of multiple dense layers [1], also called full-connected layers and we depict its structure in Supplementary Fig. 2. $\boldsymbol{\xi}$ contains trainable parameters of all layers. For every given state ρ, the input of the representation network is a pair $(\mathbf{m}_i, \mathbf{p}_i)$, where $\mathbf{m}_i$ is parameterization of a POVM measurement and $\mathbf{p}_i$ is the vector of outcome probabilities generated by performing the measurement $\mathbf{m}_i$ on the state ρ. The output $\mathbf{r}_i$ can be regarded as an abstract representation of the data $(\mathbf{m}_i, \mathbf{p}_i)$. These data are then aggregated into a single vector $\mathbf{r}$, which provides an abstract representation of the state ρ. Here, for simplicity, we just use the average function $\mathbf{r} := \frac{1}{s}\sum_{i=1}^{s}\mathbf{r}_i$ as the aggregate function. In general, more sophisticated architecture such as recurrent neural network [1] may achieve better performance, although they lead to higher requirements for hardware and hyperparameter tuning.

The structure of the representation network is the same in the training and in the test phase. When the training or test phase involves multiple quantum states, the above procedure is applied for each quantum state: for example, if the test phase involves states $(\rho^{(j)})_{j=1}^{K}$, the representation network constructs a state representation vector $\mathbf{r}^{(j)}$ for each state $\rho^{(j)}$, by aggregating the vectors associated to different measurements performed on $\rho^{(j)}$. Note that state representations of different vectors are treated separately, and are never aggregated together.

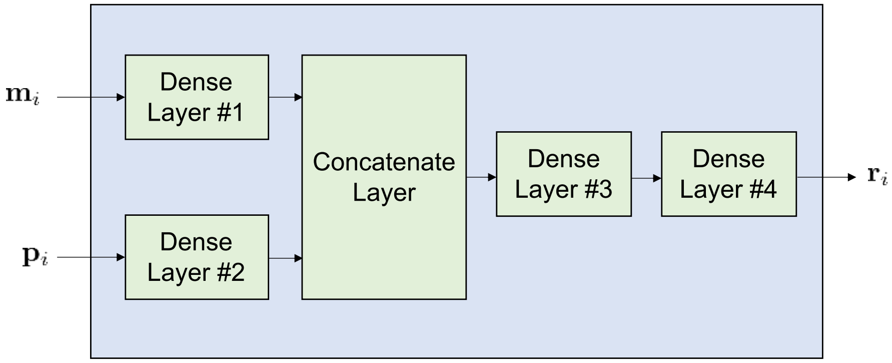

Supplementary Figure 2: Structure of the representation network.

The generation network $g_{\boldsymbol{\eta}}$ is special because its structure is different in the training and test phase. Here $\boldsymbol{\eta}$ contains all trainable parameters in the generation network. In the test phase, the generation network consists of two dense layers and one long short-term memory (LSTM) cell [2], and we depict its structure in Supplementary Fig. 3. The input of this generation network is the state representation $\mathbf{r}$ of a given quantum state ρ and the parameterization $\mathbf{m}'$ of a query POVM measurement and the output $\mathcal{N}'$ is a distribution of the prediction $\mathbf{p}'$ of measurement outcome

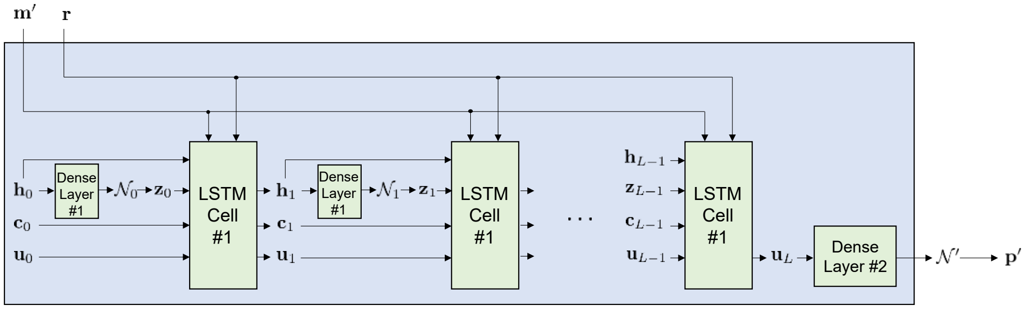

Supplementary Figure 3: Structure of the generation network in the test.

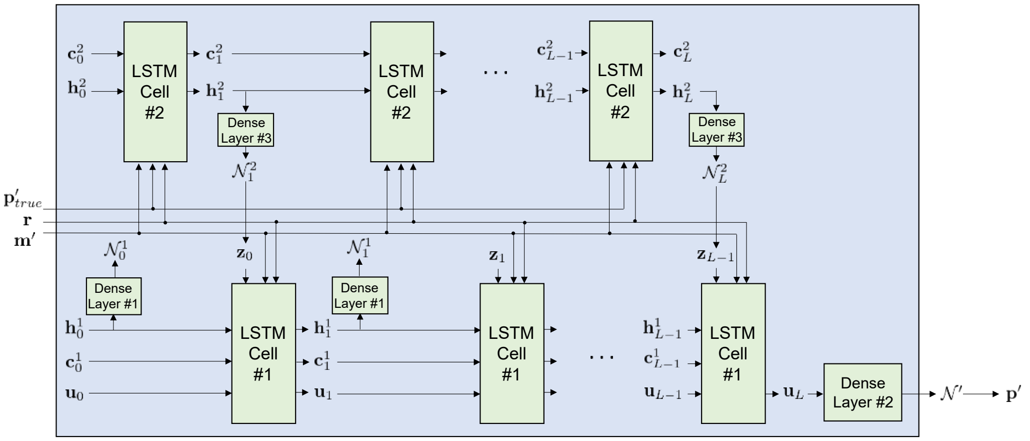

Supplementary Figure 4: Structure of the generation network in the training.

probabilities corresponding to $\mathbf{m}'$. $\mathbf{h}_0$, $\mathbf{c}_0$ and $\mathbf{u}_0$ are some internal parameters, all of which are initialized as zero tensors. As we can see, the generation network executes Dense Layer #1 and the LSTM cell for L times while $\mathbf{m}'$ and $\mathbf{r}$ are injected to the network for each time. It is worth mentioning that $\mathbf{z}_i$ ($i \in \mathbb{N}, i < L$) can be viewed as a hidden variable that obeys a prior Gaussian distribution $\mathcal{N}_i$ generated by Dense Layer #1 from $\mathbf{h}_i$. In the end, the output $\mathbf{u}_L$ of the last LSTM cell is fed into a second dense layer (Dense Layer #2) to obtain the output $\mathcal{N}'$, from which the prediction $\mathbf{p}'$ is sampled.

In the training phase, the structure of the generation network is different, and is illustrated in Supplementary Fig. 4. Here, the generation network has an extra input $\mathbf{p}'_{\text{true}}$, corresponding to the exact probability distribution for the measurement $\mathbf{m}'$ on the state ρ under consideration. Furthermore, we utilize another LSTM cell and another dense layer (Dense Layer #3) to generate a posterior distribution $\mathcal{N}_i^2$ from $\mathbf{p}'_{\text{true}}$ of the hidden variable $\mathbf{z}_i$ rather than sampling $\mathbf{z}_i$ from a prior distribution $\mathcal{N}_i^1$. The advantage of such design is that we can make good use of the information of $\mathbf{p}'_{\text{true}}$ to obtain better $\mathbf{z}_i$ during the generation. $\mathbf{h}_0^1$, $\mathbf{c}_0^1$, $\mathbf{h}_0^2$, $\mathbf{c}_0^2$ and $\mathbf{u}_0$ are some internal parameters, all of which are initialized as zero tensors.

When the training and/or test phase involve multiple states, the generation network is used to make predictions on the measurement statistics of each state, using the state representation of that state as the input. For example, when the test phase involves multiple states $\left(\rho^{(j)}\right)_{j=1}^{K}$, the generation network is given in input the measurement parametrization $\mathbf{m}'$ and the state representation $\mathbf{r}^{(j)}$ for the state $\rho^{(j)}$, and produces in output (an approximation of) the outcome probability distribution $\mathbf{p}^{(j)\prime}$ generated by the measurement $\mathbf{m}'$ on state $\rho^{(j)}$.

B. Training of GQNQ

For a given state ρ, we define the loss function $\mathcal{L}$ of the training in Eq. (1).

$$\mathcal{L}(\boldsymbol{\xi},\boldsymbol{\eta}) = \mathbb{E}[-\ln \mathcal{N}'(\mathbf{p}'_{\text{true}}) + \sum_{j=0}^{L-1} \text{KL}(\mathcal{N}_j^1, \mathcal{N}_{j+1}^2)], \tag{1}$$

where $\mathcal{N}'(\mathbf{p}'_{\text{true}})$ denotes the relative likelihood that the variable following distribution $\mathcal{N}'$ takes the value $\mathbf{p}'_{\text{true}}$, the true probability distribution generated by measurement $\mathbf{m}'$ on state ρ, and KL represents KL divergence [3] of two probability distributions. The first term of this loss function can be interpreted as the reconstruction loss, which can guide the model to acquire more accurate predictions. The second term is a regularization term utilized for seeking a better prior distribution of the hidden variable $\mathbf{z}_i$ in the generation process, which is constructive to improving the accuracy of the predictions further. When the training phase involves multiple states, we define the loss function as the sum of the function in Eq. (1) over all possible states.

We adopt batch gradient descent [4] and the Adam optimizer [5] to minimize this loss function in the training. The batch size is set to 10 or 20 in all of our experiments and the learning rate decreases gradually with the increase of the number of training epochs.

Our neural networks are implemented by the pytorch [6] framework and trained on four NVIDIA GeForce GTX 1080 Ti GPUs. The training time is less than three hours for each task discussed in this paper. The ground states of the Hamiltonian of one-dimensional Ising models utilized in our numerical experiments are solved by the exact method for the scenario of $L = 6$ and are approximately solved by density-matrix renormalization group (DMRG) [7] for the scenario of $L = 10$, 20 and 50. The data of continuous-variable quantum states are generated by simulation tools provided in Strawberry Fields [8].

We present the whole training procedure by pseudocode in Algorithm 1 following the notations introduced in main text.

Algorithm 1: Training of generative query network for quantum state learning.

Data: number of states in training set N, state measurement results $\{\{(\mathbf{m}_i, \mathbf{p}_i^k)\}_{i=1}^n\}_{k=1}^N$, maximum number of known POVM measurement results for each state a, maximum number of epochs E, learning rate δ, batch size B.

Initialize parameters $\boldsymbol{\xi}$ and $\boldsymbol{\eta}$ randomly, $e = 0$;

while $e < E$ **do**
- $\mathcal{L} = 0$;
- **for** $k = 1$ **to** N **do**
  - Generate a random integer number n_1 from $[1, a]$;
  - Randomly select n_1 pairs of $(\mathbf{m}_i, \mathbf{p}_i^k)$ from $\{(\mathbf{m}_i, \mathbf{p}_i^k)\}_{i=1}^n$ and denote them as $\{(\mathbf{m}_{i_j}, \mathbf{p}_{i_j}^k)\}_{j=1}^{n_1}$, where $\{i_j\}_{j=1}^n$ is a permutation of $\{1, \ldots, n\}$;
  - Input each of $\{(\mathbf{m}_{i_j}, \mathbf{p}_{i_j}^k)\}_{j=1}^{n_1}$ into the representation network $f_{\boldsymbol{\xi}}$ to obtain the representations $\{\mathbf{r}_{i_j}\}_{j=1}^{n_1}$ as $\mathbf{r}_{i_j} = f_{\boldsymbol{\xi}}(\mathbf{m}_{i_j}, \mathbf{p}_{i_j}^k)$;
  - Calculate the state representation by an aggregate function $\mathcal{A}$ as $\mathbf{r} = \mathcal{A}(\{\mathbf{r}_{i_j}\}_{j=1}^{n_1})$;
  - Input $\mathbf{r}$ and the remaining $\{\mathbf{m}_{i_j}\}_{j=n_1+1}^n$ into the generation network $g_{\boldsymbol{\eta}}$ to obtain the predictions $\{\mathbf{p}_{i_j}'^k\}_{j=n_1+1}^n$ of measurement outcome distributions as $\mathbf{p}_{i_j}'^k = g_{\boldsymbol{\eta}}(\mathbf{r}, \mathbf{m}_{i_j})$;
  - Calculate the loss l with Eq. (1) by comparing $\{\mathbf{p}_{i_j}'^k\}_{j=n_1+1}^n$ with $\{\mathbf{p}_{i_j}^k\}_{j=n_1+1}^n$ and update $\mathcal{L}$ as $\mathcal{L} = \mathcal{L} + l$;
  - **if** k *mod* $B = 0$ **then**
    - Calculate $\nabla_{\boldsymbol{\xi}}\mathcal{L}$ and $\nabla_{\boldsymbol{\eta}}\mathcal{L}$;
    - Update $\boldsymbol{\xi}$ and $\boldsymbol{\eta}$ as $\boldsymbol{\xi} = \boldsymbol{\xi} - \delta\nabla_{\boldsymbol{\xi}}\mathcal{L}$, $\boldsymbol{\eta} = \boldsymbol{\eta} - \delta\nabla_{\boldsymbol{\eta}}\mathcal{L}$;
    - $\mathcal{L} = 0$;
- $e = e + 1$;

C. Details of Experiments

a. Datasets. In the experiments for ground states of Ising models and XXZ models, the training set is composed of 40 different states for each J or Δ while the test set is composed of 10 different states for each J or Δ. As for the experiments for GHZ state with local rotation and W state with local rotation, we generate 800 states for training and 200 states for test. In the experiments for continuous-variable quantum states, we randomly generate 10000 different cat states, gaussian states and GKP states and split them into training and test sets with $4 : 1$ ratio.

Supplementary Table I: Average classical fidelity between predicted outcome statistics and real outcome statistics, averaged over all the test states and random query measurements. The eight rows correspond to eight different scenarios, where GQNQ is trained and tested over measurement data of nine sets of states. The values of $d_{\mathbf{r}}$, $d_{\mathbf{h}}$ and $d_{\mathbf{z}}$ are different for each column.

Types of states	$d_{\mathbf{r}}=2$, $d_{\mathbf{h}}=2$, $d_{\mathbf{z}}=2$	$d_{\mathbf{r}}=2$, $d_{\mathbf{h}}=6$, $d_{\mathbf{z}}=2$	$d_{\mathbf{r}}=4$, $d_{\mathbf{h}}=12$, $d_{\mathbf{z}}=4$	$d_{\mathbf{r}}=8$, $d_{\mathbf{h}}=24$, $d_{\mathbf{z}}=8$	$d_{\mathbf{r}}=16$, $d_{\mathbf{h}}=48$, $d_{\mathbf{z}}=16$	$d_{\mathbf{r}}=32$, $d_{\mathbf{h}}=96$, $d_{\mathbf{z}}=32$
(i) Ising ground states with ferromagnetic bias	0.7987	0.8835	0.8981	0.9255	0.9543	0.9870
(ii) Ising ground states with antiferromagnetic bias	0.7896	0.8739	0.8894	0.9236	0.9562	0.9869
(iii) Ising ground states with no bias	0.7999	0.8911	0.8993	0.9277	0.9596	0.9895
(iv) XXZ ground states with ferromagnetic bias	0.6386	0.7683	0.9038	0.9546	0.9603	0.9809
(v) XXZ ground states with XY phase bias	0.7515	0.8102	0.8359	0.8924	0.9352	0.9601
(vi) (i)-(v) together	0.7143	0.7709	0.8376	0.8739	0.9178	0.9567
(vii) GHZ state with local rotations	0.8342	0.8816	0.9271	0.9502	0.9579	0.9744
(viii) W state with local rotations	0.9249	0.9310	0.9579	0.9733	0.9771	0.9828
(ix) (i)-(v), (vii) and (viii) together	0.6936	0.7685	0.8369	0.8725	0.9085	0.9561

b. Number of trainable parameters. We mainly adopt three kinds of models for three different tasks. In the experiments for learning discrete quantum states, we exploit the models with 6676544 trainable parameters for the scenario of $L = 6$ while exploiting the models with 45484 trainable parameters for the scenario of $L = 10, 20$ and 50. In the experiments for learning continuous-variable quantum states, we exploit the models with 35572 trainable parameters.

c. Maximum number of known POVM measurement results for each state in the training. We set maximum number of known POVM measurement results for each state a in the training as 200 for the six-qubit cases, 50 for the 10-, 20- and 50-qubit cases and 150 for the cases of continuous states.

d. Initialization and learning rate. For each task, we initialize the parameters of the models randomly before the training. The learning rate is set as 0.01 initially and decreases as the number of iterations increases.

e. Number of epochs and training time. We usually set the maximum number of epochs E as 200 and the batch size B as 30 in the training. The training time varies with the size of training set for each task while the training time is always less than three hours in all of the experiments.

Supplementary Note 2 Hyperparameters

As introduced above, there are some hyperparameters in our GQNQ model and the most significant ones are the dimensions of $\mathbf{r}_i$, $\mathbf{h}_i$ and $\mathbf{z}_i$, because they affect the size of the state representation and the complexity of the model, and thus affect the performance of the model. We denote them as $d_{\mathbf{r}}$, $d_{\mathbf{h}}$ and $d_{\mathbf{z}}$ respectively. In this section, we conduct a series of experiments to explore how the choice of hyperparameters affects the performance of the proposed model. We take the settings of learning 6-qubit states introduced in the main text as examples. In each experiment, different settings of hyperparameters are adopted and the results are shown in Supplementary Table I.

We can easily find that as the complexity of the model increases, the performance of the model becomes better. However, it must be pointed out that the complexity of the model cannot be arbitrarily high considering the memory size and the difficulty of training, and the models with $d_{\mathbf{r}} = 32$, $d_{\mathbf{h}} = 96$ and $d_{\mathbf{z}} = 32$ are the most complicated ones we consider in this paper.

Supplementary Note 3 Arbitrary State Learning

Furthermore, we conducted experiments to learn arbitrary 6-qubit quantum states and the results are shown in Supplementary Table II. We claim that all models failed in this case, since we find that they always yield distributions close to the uniform distribution for any query measurement, which means that the model cannot learn an effective state representation to generate accurate measurement outcome statistics. A possible explanation is that the model is not complicated enough to handle an unstructured, highly complex dataset. Although a more complicated model might be more effective intuitively, such a model may require larger training set and be less efficient. As expected, our GQNQ model is designed for quantum states sharing a common structure and is not suitable for arbitrary quantum states.

Supplementary Table II: Average classical fidelity between predicted outcome statistics and real outcome statistics for the arbitrary states, averaged over all the test states and random query measurements. The values of $d_{\mathbf{r}}$, $d_{\mathbf{h}}$ and $d_{\mathbf{z}}$ are different for each column.

Types of states	Uniform distribution	$d_{\mathbf{r}}=2,\ d_{\mathbf{h}}=2,\ d_{\mathbf{z}}=2$	$d_{\mathbf{r}}=2,\ d_{\mathbf{h}}=6,\ d_{\mathbf{z}}=2$	$d_{\mathbf{r}}=4,\ d_{\mathbf{h}}=12,\ d_{\mathbf{z}}=4$	$d_{\mathbf{r}}=8,\ d_{\mathbf{h}}=24,\ d_{\mathbf{z}}=8$	$d_{\mathbf{r}}=16,\ d_{\mathbf{h}}=48,\ d_{\mathbf{z}}=16$	$d_{\mathbf{r}}=32,\ d_{\mathbf{h}}=96,\ d_{\mathbf{z}}=32$
Arbitrary 6-qubit state	0.8879	0.8879	0.8879	0.8879	0.8879	0.8879	0.8879

Supplementary Note 4   Generalization from Informationally Incomplete Measurements

In this section, we will further discuss the generalization performance of our proposed model in the examples of six-qubit quantum states. We mainly focus on how the information completeness of the measurement class affects the final performance. Rather than setting the class of measurements $\mathcal{M}$ as the set of all 729 six-qubit Pauli-basis measurements, we construct $\mathcal{M}$ by randomly selecting 72 different six-qubit Pauli-basis measurements in each experiment here. For each dataset we discussed, we did such experiments and averaged the results. The results are shown in Supplementary Table III.

As the experimental results show, our proposed model still has a satisfactory performance when the measurement class is not informationally complete. Meanwhile, we also find that the model will generalize worse as the complexity of datasets increases. A possible explanation is that more information is needed to yield accurate state representations when the dataset is composed of multiple types of states.

Supplementary Table III: Average classical fidelity between predicted outcome statistics and real outcome statistics, averaged over all the test states and random query measurements. The measurement class $\mathcal{M}$ is composed of 72 different six-qubit Pauli-basis measurements in the case of informationally incomplete measurements.

Types of states	Informationally complete $\mathcal{M}$	Informationally incomplete $\mathcal{M}$
(i) Ising ground states with ferromagnetic bias	0.9870	0.9865
(ii) Ising ground states with antiferromagnetic bias	0.9869	0.9863
(iii) Ising ground states with no bias	0.9895	0.9812
(iv) XXZ ground states with ferromagnetic bias	0.9809	0.9713
(v) XXZ ground states with XY phase bias	0.9601	0.9495
(vi) (i)-(v) together	0.9567	0.9447
(vii) GHZ state with local rotations	0.9744	0.9694
(viii) W state with local rotations	0.9828	0.9824
(ix) (i)-(v), (vii) and (viii) together	0.9561	0.9442

We also study the generalization performances of GQNQ for continuous-variable states when $\mathcal{M}$ is information incomplete over the truncated subspace of interest (less than 30 photons). In the main text, $\mathcal{M}$ consists of 300 homodyne measurements with equidistant phases θ and is informationlly complete over the truncated subspace with less than 300 photons [9]. In contrast, here we test the scenario where $\mathcal{M}$ consists of only 10 homodyne measurement settings with phases $\theta \in \{0, \pi/10, \ldots, 9\pi/10\}$, and $\mathcal{S}$ is subset of $\mathcal{M}$ containing 5 random homodyne measurement settings. Note that now $\mathcal{M}$ is insufficient to fully characterize a density matrix on a truncated subspace with more than nine photons. We train and test GQNQ using data from three types of continuous-variable states as discussed in the main text in this scenario. The average and worst classical fidelities over all query measurements, together with the comparison with the scenario where $|\mathcal{M}| = 300$ and $|\mathcal{S}| = 10$, are presented in Supplementary Table IV. The results show that even in this information incomplete scenario, GQNQ still shows great prediction performance on all the types of test states. Again this is because the states we consider fall within lower-dimensional corners of the subspace with limited photons.

Supplementary Note 5   Overfitting

Overfitting due to unbalanced data can be an important issue when GQNQ is used for learning across multiple types of states. Here we study the six-qubit scenario where GQNQ is trained and tested on the union of the datasets of ground states of Ising model, ground states of XXZ model, GHZ states and W states with local rotations. Specifically, one type of states is chosen to be underrepresented, appearing 10 times less frequently than any other type of

Supplementary Table IV: Generalization performances of GQNQ on continuous-variable quantum states.

Type of states for training and test	$\lvert\mathcal{M}\rvert = 300$ (Avg)	$\lvert\mathcal{M}\rvert = 300$ (Worst)	$\lvert\mathcal{M}\rvert = 10$ (Avg)	$\lvert\mathcal{M}\rvert = 10$ (Worst)
(i) Squeezed thermal states	0.9973	0.9890	0.9953	0.9901
(ii) Cat states	0.9827	0.9512	0.9571	0.8920
(iii) GKP states	0.9762	0.9405	0.9633	0.9470
(iv) (i)-(iii) together	0.9658	0.9077	0.9507	0.8843

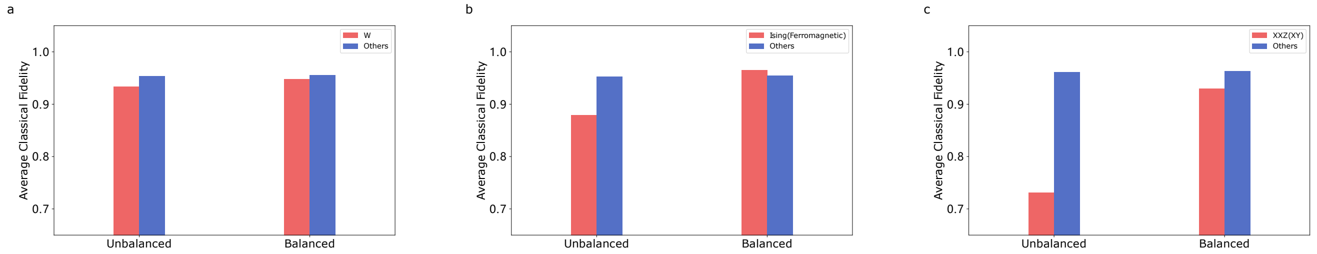

Supplementary Figure 5: Performances of GQNQ when training data from different types of quantum states are unbalanced. In each figure, the red bar represents the classical fidelity with respect to the chosen underrepresented type of states, and the blue bar represents the classical fidelity averaged over all other types of states. Fig.(a) compares the average classical fidelity for W states with local rotations and the average classical fidelity for all other states when the ratio of the size of training data from W states to any other type is $1:10$ and $1:1$, respectively. Fig.(b) compares the average classical fidelity for ground states of ferromagnetic Ising model with the average classical fidelity for all other states when the ratio of the size of training data from ferromagnetic Ising model to any other type is $1:10$ and $1:1$, respectively. Fig.(c) compares the average classical fidelity for ground states of XXZ model in XY phase with the average classical fidelity for all other states when the ratio of the size of training data from XXZ model in XY phase to any other type is $1:10$ and $1:1$, respectively.

states in the whole training dataset. Then we test the prediction performances of GQNQ with respect to both this underrepresented type of states and the other types of states as shown in Supplementary Fig. 5.

The results show that the performance of GQNQ with unbalanced training data depends on the state under consideration. For W states with local rotations we find that unbalanced training data has little effect on the performance. The situation is similar for the ground states of the Ising model in the ferromagnetic phase. In contrast, the prediction for XXZ model in the XY phase drops to 0.73 when the training data are unbalanced. The results agree with the phenomenon that the ground states of XXZ model in the XY phase are more difficult to learn than any other type of states we considered.

Supplementary Note 6 Additional Experiments

A. Ising model

We study the performance of GQNQ for 10-, 20- and 50-qubit Ising ground states when the measurements are nearest-neighbour two-qubit Pauli measurements. Different from the setting in the main text, here we choose J_i as a Gaussian variable with mean value J and variance 0.01. Hence when J is around 0, both ferromagnetic interactions and antiferromagnetic interactions are present with high probability. We find that GQNQ cannot give good predictions of outcome statistics in this scenario when both ferromagnetic and antiferromagnetic interactions exist. The results, together with the comparison with the scenario where each J_i is chosen to be the absolute value (or the opposite of the absolute value, for $J < 0$) of the Gaussian variable, are presented in Supplementary Fig. 6.

B. Cat states

For the numerical experiments on learning of continuous-variable quantum states, we provide an example of comparison between predictions and ground truths for a cat state in Supplementary Fig. 7 here.

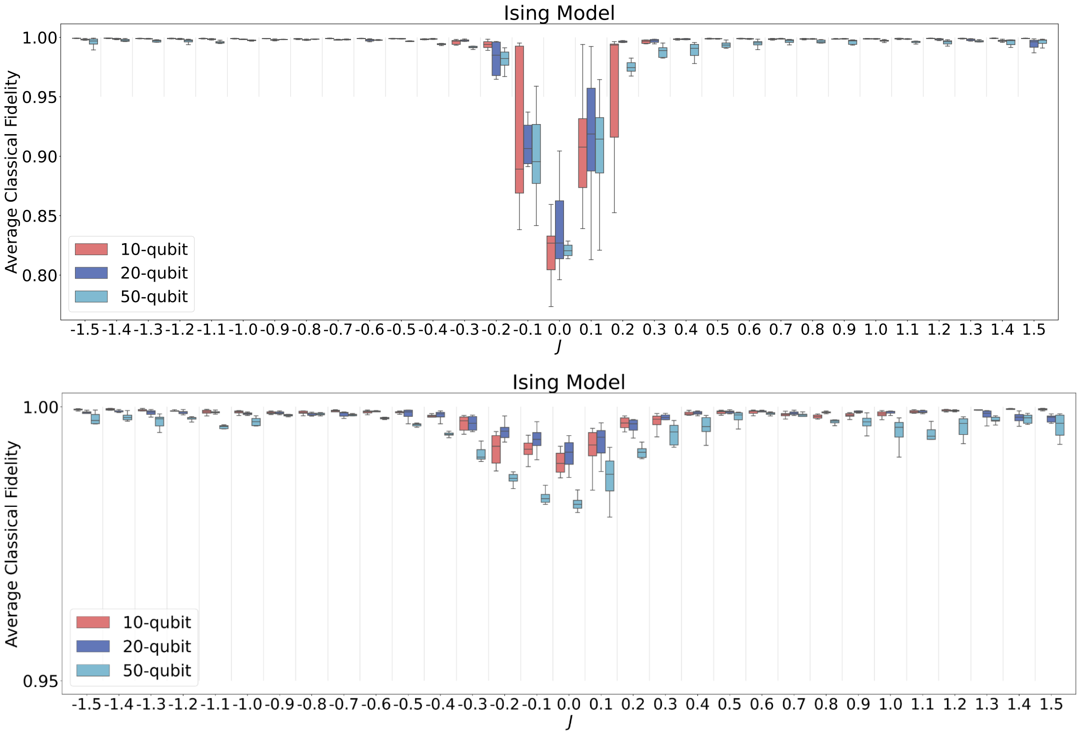

Supplementary Figure 6: Comparison between the performances of GQNQ in Ising model when both ferromagnetic and antiferromagnetic interactions are present near $J = 0$ (top) vs when only either ferromagnetic or antiferromagnetic interactions are present near $J = 0$ (bottom).

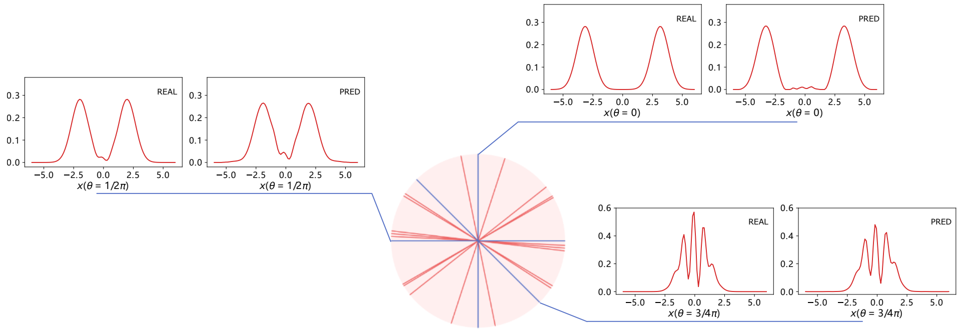

Supplementary Figure 7: The true outcome probability density (left) and the predicted probability density (right) for cat state $|2.22 + 1.41\mathrm{i}, \pi/4\rangle_{\mathrm{cat}}$ at quadrature phases $\theta = 0$, $\theta = \pi/2$ and $\theta = 3\pi/4$, respectively, given the measurement outcome densities at ten random quadrature phases. In the middle circle, ten red lines passing through the center represent those quadrature phases at which measurement outcome statistics are known, and three blue lines passing through the center represent those quadrature phases at which measurement outcome statistics are to be predicted.

Supplementary Note 7 Training with data from the state to be characterized

In this section, we discuss how to train our GQNQ model with data only from the quantum state to be characterized. In this setting, GQNQ behaves as a completely unsupervised learner that predicts the outcome statistics of unperformed measurements using measurement data obtained from the quantum state under consideration. The set $\mathcal{M}_*$ of fiducial measurements in the training coincides with the set $\mathcal{S}$ of performed measurements. In the training, GQNQ is trained with $s (s < n)$ measurement results $\{(\mathbf{m}_i, \mathbf{p}_i)\}_{i=1}^{s}$ corresponding to $\mathcal{S}$. When the training is finished, the trained model can be utilized to predict the outcome statistics corresponding to $\mathcal{M} \setminus \mathcal{S}$.

We present the whole training procedure in such setting by pseudocode in Algorithm 2.

Algorithm 2: Training of GQNQ with data provided from the quantum state to be characterized.

Data: State measurement results $\{(\mathbf{m}_i, \mathbf{p}_i)\}_{i=1}^{s}$ of the quantum state to be characterized corresponding to the set of reference measurements $\mathcal{M}_*$, maximum number of known POVM measurement results $a (a < s)$ in the training, maximum number of epochs E, learning rate δ.

Initialize parameters $\boldsymbol{\xi}$ and $\boldsymbol{\eta}$ randomly, $e = 0$;

while $e < E$ **do**

- $\mathcal{L} = 0$;
- Generate a random integer number n_1 from $[1, a]$;
- Randomly select n_1 pairs of $(\mathbf{m}_i, \mathbf{p}_i)$ from $\{(\mathbf{m}_i, \mathbf{p}_i)\}_{i=1}^{s}$ and denote them as $\{(\mathbf{m}_{i_j}, \mathbf{p}_{i_j})\}_{j=1}^{n_1}$, where $\{i_j\}_{j=1}^{s}$ is a permutation of $\{1, \ldots, s\}$;
- Input each of $\{(\mathbf{m}_{i_j}, \mathbf{p}_{i_j})\}_{j=1}^{n_1}$ into the representation network $f_{\boldsymbol{\xi}}$ to obtain the representations $\{\mathbf{r}_{i_j}\}_{j=1}^{n_1}$ as $\mathbf{r}_{i_j} = f_{\boldsymbol{\xi}}(\mathbf{m}_{i_j}, \mathbf{p}_{i_j})$;
- Calculate the state representation by an aggregate function $\mathcal{A}$ as $\mathbf{r} = \mathcal{A}(\{\mathbf{r}_{i_j}\}_{j=1}^{n_1})$;
- Input $\mathbf{r}$ and the remaining $\{\mathbf{m}_{i_j}\}_{j=n_1+1}^{s}$ into the generation network $g_{\boldsymbol{\eta}}$ to obtain the predictions $\{\mathbf{p}'_{i_j}\}_{j=n_1+1}^{s}$ of measurement outcome distributions as $\mathbf{p}'_{i_j} = g_{\boldsymbol{\eta}}(\mathbf{r}, \mathbf{m}_{i_j})$;
- Calculate the loss l with Eq. (1) by comparing $\{\mathbf{p}'_{i_j}\}_{j=n_1+1}^{s}$ with $\{\mathbf{p}_{i_j}\}_{j=n_1+1}^{s}$ and update $\mathcal{L}$ as $\mathcal{L} = \mathcal{L} + l$;
- Calculate $\nabla_{\boldsymbol{\xi}}\mathcal{L}$ and $\nabla_{\boldsymbol{\eta}}\mathcal{L}$;
- Update $\boldsymbol{\xi}$ and $\boldsymbol{\eta}$ as $\boldsymbol{\xi} = \boldsymbol{\xi} - \delta\nabla_{\boldsymbol{\xi}}\mathcal{L}$, $\boldsymbol{\eta} = \boldsymbol{\eta} - \delta\nabla_{\boldsymbol{\eta}}\mathcal{L}$;
- $\mathcal{L} = 0$;
- $e = e + 1$;

Supplementary References

[1] Charu C Aggarwal *et al.*, "Neural networks and deep learning," Springer **10**, 978–3 (2018).
[2] Sepp Hochreiter and Jürgen Schmidhuber, "Long short-term memory," Neural Comput. **9**, 1735–1780 (1997).
[3] Solomon Kullback, *Information theory and statistics* (Courier Corporation, 1997).
[4] Sebastian Ruder, "An overview of gradient descent optimization algorithms," arXiv preprint arXiv:1609.04747 (2016).
[5] Diederik P Kingma and Jimmy Ba, "Adam: A method for stochastic optimization," arXiv preprint arXiv:1412.6980 (2014).
[6] Adam Paszke, Sam Gross, Francisco Massa, Adam Lerer, James Bradbury, Gregory Chanan, Trevor Killeen, Zeming Lin, Natalia Gimelshein, Luca Antiga, *et al.*, "Pytorch: An imperative style, high-performance deep learning library," Adv. Neural. Inf. Process. Syst. **32**, 8026–8037 (2019).
[7] Ulrich Schollwöck, "The density-matrix renormalization group," Rev. Mod. Phys. **77**, 259 (2005).
[8] Nathan Killoran, Josh Izaac, Nicolás Quesada, Ville Bergholm, Matthew Amy, and Christian Weedbrook, "Strawberry fields: A software platform for photonic quantum computing," Quantum **3**, 129 (2019).
[9] Ulf Leonhardt, *Measuring the quantum state of light*, Vol. 22 (Cambridge university press, 1997).
